# Clinical Spectrum of Tauopathies

**DOI:** 10.3389/fneur.2022.944806

**Published:** 2022-07-14

**Authors:** Nahid Olfati, Ali Shoeibi, Irene Litvan

**Affiliations:** ^1^Department of Neurology, Faculty of Medicine, Mashhad University of Medical Sciences, Mashhad, Iran; ^2^UC San Diego Department of Neurosciences, Parkinson and Other Movement Disorder Center, San Diego, CA, United States

**Keywords:** tauopathy, movement, clinical, progressive supranuclear palsy, corticobasal, neurodegenerative, frontotemporal dementia, primary progressive aphasia

## Abstract

Tauopathies are both clinical and pathological heterogeneous disorders characterized by neuronal and/or glial accumulation of misfolded tau protein. It is now well understood that every pathologic tauopathy may present with various clinical phenotypes based on the primary site of involvement and the spread and distribution of the pathology in the nervous system making clinicopathological correlation more and more challenging. The clinical spectrum of tauopathies includes syndromes with a strong association with an underlying primary tauopathy, including Richardson syndrome (RS), corticobasal syndrome (CBS), non-fluent agrammatic primary progressive aphasia (nfaPPA)/apraxia of speech, pure akinesia with gait freezing (PAGF), and behavioral variant frontotemporal dementia (bvFTD), or weak association with an underlying primary tauopathy, including Parkinsonian syndrome, late-onset cerebellar ataxia, primary lateral sclerosis, semantic variant PPA (svPPA), and amnestic syndrome. Here, we discuss clinical syndromes associated with various primary tauopathies and their distinguishing clinical features and new biomarkers becoming available to improve *in vivo* diagnosis. Although the typical phenotypic clinical presentations lead us to suspect specific underlying pathologies, it is still challenging to differentiate pathology accurately based on clinical findings due to large phenotypic overlaps. Larger pathology-confirmed studies to validate the use of different biomarkers and prospective longitudinal cohorts evaluating detailed clinical, biofluid, and imaging protocols in subjects presenting with heterogenous phenotypes reflecting a variety of suspected underlying pathologies are fundamental for a better understanding of the clinicopathological correlations.

## Introduction

Tauopathies are both clinical and pathological heterogenous disorders characterized by neuronal and/or glial accumulation of misfolded tau protein. Since the first appearance of the name “tau protein” in the literature (1), many studies have been conducted to explain its structure and function. A larger and expanding body of literature focuses on untangling the process of tau accumulation in the neural tissue. Tau is a microtubule-associated protein translated from the MAPT gene on chromosome 17q21 which is predominantly expressed in the brain and the skeletal muscles (2). Alternate splicing of three exons of the MAPT gene produces two sets of isoforms with either three or four microtubule-binding domains, namely 3R and 4R isoforms (3). Based on the ratio of the 3R to 4R isoforms in the pathologic aggregates, tauopathies could be pathologically classified as 4R, 3R, or mixed 3R and 4R. Table 1 shows the pathologic spectrum of tauopathies based on 3R:4R ratio in tau aggregates. Although cryo-electron microscopic (cryo-EM) evaluation of tau filaments in tauopathies led to a new classification based on the structure of tau filaments (16), the clinical significance of this classification is not yet known. There is also a distinction between primary vs. secondary tauopathies based on the tauopathy being the predominant or merely a co-pathology. In fact, even in many secondary tauopathies, tau is the most important co-pathology and a critical player in the process of neurodegeneration (17, 18). It is now well understood that every pathologic tauopathy may present with various clinical phenotypes based on the primary site of involvement and the spread and distribution of the pathology in the nervous system (19). Thus, clinicopathological correlation is becoming more and more challenging and defining clinical diagnostic criteria for pathologic disorders more nuanced and sometimes almost impossible.

**Table 1:** Pathologic spectrum of primary tauopathies and their defining hallmarks.

	**4R tauopathies**					**3R tauopathy**	**Mixed 3R/4R tauopathy**
**Lesion**	**PSP (4, 5)**	**CBD (5, 6)**	**GGT (7, 8)**	**AGD (9, 10)**	**ARTAG (11, 12)**	**PiD (13, 14)**	**PART (15)**
Neuronal loss/gliosis	Frontal / paracentral Globus pallidus Subthalamic nucleus Substantia nigra Brainstem tegmentum Red nucleus Midbrain tectum Dentate nucleus	Asymmetric peri-rolandic areas, superior frontal and parietal Substantia nigra	Frontotemporal (type I, III) Motor cortex / corticospinal tract (types II, III)	Ambient gyrus medial temporal structures (CA1)	Subpial, subependymal, perivascular in basal or lobar regions	Frontal, anterior temporal, medial temporal Subcortical/ white matter degeneration	Medial temporal Hippocampal formation (CA2)
Neuronal pathology/inclusions	Dense argyrophilic “globose” tangles	Disperse globose inclusions (less argyrophilic mostly consisted of pre-tangles) Abundant achromatic /ballooned neurons	Diffuse granular /thick cord-like /round horseshoe-shaped neuronal cytoplasmic inclusions	Comma-shaped argyrophilic grains Cytoplasmic pre-tangles Ballooned neurons		Strongly argyrophilic neuronal cytoplasmic inclusions (Pick bodies) Pick cells (ballooned achromatic neurons)	Intracellular neurofibrillary tangles Extracellular “ghost” tangles
Glial pathology/ inclusions	Tufted astrocytes (highly argyrophilic dense NFTs in soma)	Astrocytic plaques (less argyrophilic NFTs in processes)	Globular oligodendroglial / astrocytic inclusions Non-argyrophilic tufted astrocytes-like inclusions	Bush-like astrocytes	Thorn-shaped, granular or fuzzy astrocytes	“Ramified” astrocytes	
Threads / coiled bodies	Coiled bodies Threads	Coiled bodies Widespread threads	Coiled bodies	Coiled bodies			

*AGD, argyrophilic grain disease; ARTAG, aging related tau astrogliopathy; CA, cornu Ammonis; CBD, corticobasal degeneration; PSP, progressive supranuclear palsy; GGT, globular glial tauopathy; NFT, neurofibrillary tangle; PART, primary age related tauopathy; PiD, Pick's disease*.

**Figure 1:**
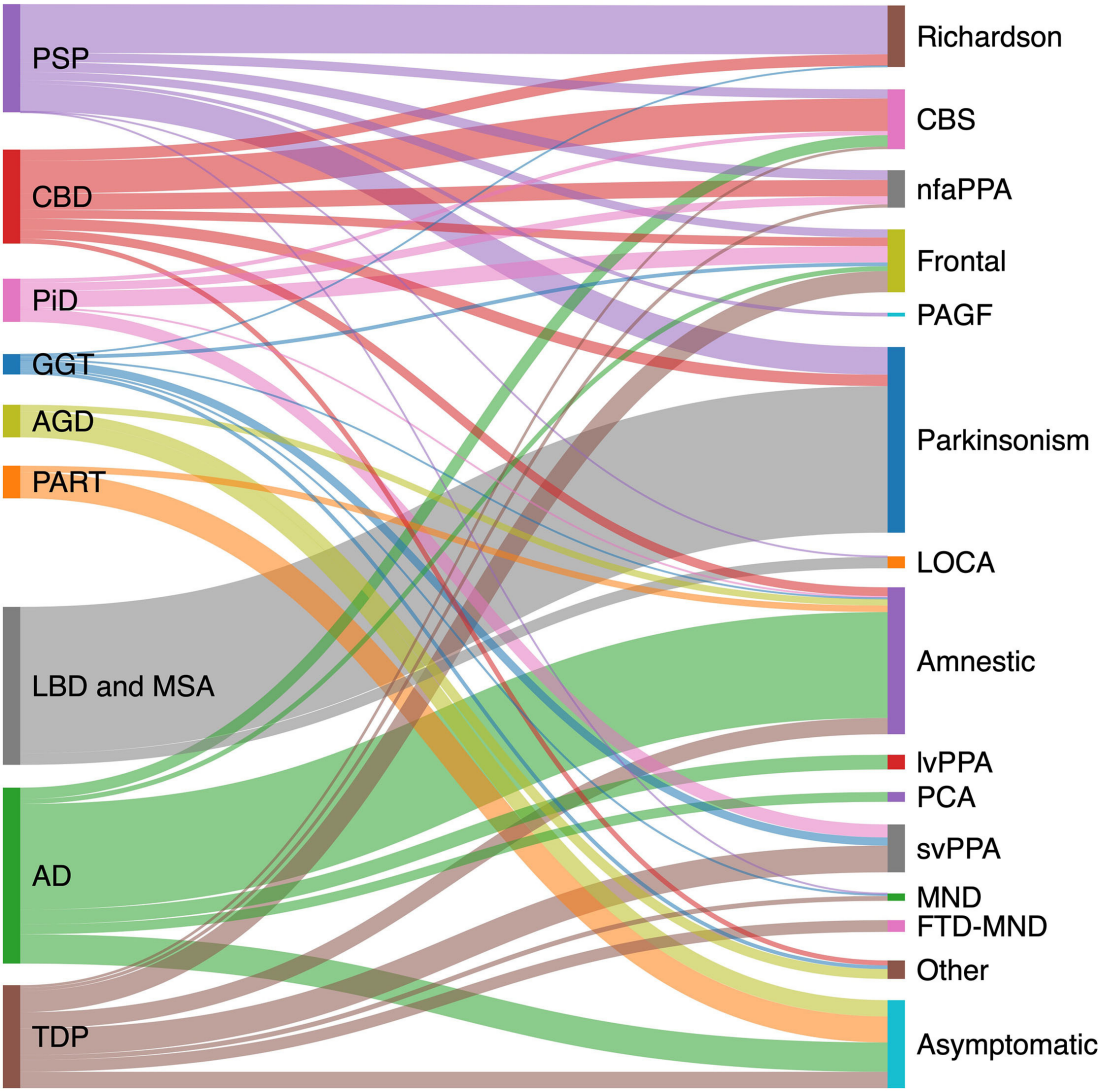
Clinicopathological correlation in tauopathies spectrum. Pathologic diagnoses are shown on the left and clinical syndromes on the right. Degree to which various pathologies contribute to a specific clinical phenotype is estimated based on available pathology-confirmed studies, including: PSP (20–23); CBD (23–27); AD (28–31); PiD (32); LBD (33); GGT (34); TDP (35–37); AGD (38); and PART (15, 39, 40). AD, Alzheimer's disease; AGD, argyrophilic grain disease; CBD, corticobasal degeneration; CBS corticobasal syndrome; FTD-MND, frontotemporal dementia-motor neuron disease; GGT, globular glial tauopathy; LBD, Lewy body disease; LOCA, late onset cerebellar ataxia; lvPPA logopenic variant primary progressive aphasia; MND, motor neuron disease; nfaPPA, non-fluent agrammatic primary progressive aphasia; PAGF, progressive akinesia and gait freezing; PART, primary age-related tauopathy; PCA, posterior cortical atrophy; PiD, Pick's disease; PSP, progressive supranuclear palsy; svPPA, semantic variant primary progressive aphasia; TDP, transactive response DNA binding protein 43 kDa pathology.

The pathologic spectrum of primary tauopathies include progressive supranuclear palsy (PSP), corticobasal degeneration (CBD), Pick's disease (PiD), globular glial tauopathy (GGT), argyrophilic grain disease (AGD), primary age-related tauopathy (PART), and aging-related tau astrogliopathy (ARTAG). On the other hand, the clinical spectrum of tauopathies include some major syndromes strongly associated with an underlying primary tauopathy and a number of other syndromes with lower specificity. The first group includes PSP or Richardson syndrome (RS), corticobasal syndrome (CBS), non-fluent agrammatic primary progressive aphasia (nfaPPA)/apraxia of speech, pure akinesia with gait freezing (PAGF), and behavioral variant frontotemporal dementia (bvFTD). The second group with less specificity includes Parkinsonian syndrome, late onset cerebellar ataxia, primary lateral sclerosis, semantic variant PPA (svPPA), and amnestic syndrome of the hippocampal type. Figure 1 shows the clinicopathologic correlation in the tauopathy spectrum. Of all clinical variants, RS has the most consistent correlation with an underlying tau, more specifically 4R tau, pathology (Figure 1). Here, we summarize main clinical syndromes associated with various primary tauopathies.

## Clinical Syndromes With Strong Association to a Primary Tauopathy

### Richardson Syndrome

Progressive supranuclear palsy (PSP) syndrome was first described by Steele, Richardson, and Olszewski in 1964 as a distinct clinicopathological entity (41). Although typical PSP pathology with tufted astrocytes (Table 1) is the most common underlying pathology of this clinical syndrome (PSP-RS), a small subset of patients with an underlying CBD pathology might present with RS (CBD-RS) and there are rare reports of GGT presenting with a PSP syndrome (Figure 1) (34, 42). Original description of RS included nine patients presenting with early and prominent supranuclear ophthalmoplegia, severe dysarthria, axial rigidity with extensor posturing of the neck, mild dementia, and pseudobulbar palsy with subsequent progression to a bedridden state and death in 5 to 7 years (41). Patients also showed gait problems with frequent falls and unsteadiness which originally were attributed by authors to the gaze palsy and truncal rigidity or truncal apraxia. However, postural instability was later acknowledged as the major cause of gait impairment in these patients (43) and as the second clinical hallmark of RS, along with supranuclear gaze palsy (44, 45).

Before recognition of the various clinical phenotypes, the prevalence of PSP has been estimated to be about 5–8/100,000 (46–48). This figure in fact might better reflect the prevalence of PSP-RS which accounts for about 40–50% of patients with PSP (20, 21). In a more recent study by Stang et al. (49), the new MDS-PSP criteria for PSP was used which led to an estimated PSP incidence of 2.6 per 100,000. Median age of the onset of RS is about 63 years (50). Survival has not changed since the first description of RS.

#### Clinical Characteristics

Ocular manifestations in RS are diverse and include eye movement, fixation, lid function, and pupillary abnormalities (51). Supranuclear ophthalmoplegia starts with subtle slowing and decreased accuracy of vertical, especially downward saccades which is better demonstrated on optokinetic nystagmus (OKN) testing (52). This will later be followed by decreased saccadic amplitude and eventual involvement of horizontal saccades (53) and smooth pursuit system (54) leading to complete ophthalmoplegia. Fixation impairment with frequent high amplitude square wave jerks (55, 56) is a differentiating features of RS from similar parkinsonian syndromes especially CBS (56). Decreased blink rate, lid retraction, blepharospasm, and eyelid opening apraxia are other ocular manifestations of RS.

Postural instability leading to frequent falls is a common feature among various Parkinsonian disorders. However, it is most severe and occurs the earliest in the course of RS, in the first 3 years and more specifically in the first year of disease onset (45), compared to other Parkinsonian syndromes and other PSP variants (57). On stance, patients with RS assume a wide base and tend to drift backwards (44, 58) and postural reflexes are impaired on the pull test. During gait initiation, the center of pressure changes in opposite direction in these patients compared to patients with Parkinson's disease (PD) and healthy controls (59). Walking in RS is markedly slow with decreased cadence and has been described as “reckless” or like a “drunken sailor,” emphasizing unsteadiness and increased variability. Increased double support time and the wide base are compensatory strategies to improve balance (60); however, despite these strategies, patients with RS are very prone to falls following minor internal or external perturbations. In addition to the aforementioned abnormalities, other factors, such as vertical supranuclear gaze palsy, bradykinesia, axial rigidity, and cognitive impairment have been proposed as contributors to the fall risk. However, a large recent study showed that vertical supranuclear gaze palsy and cognitive impairment were not independent predictors of fall risk while markers of disease duration and severity, such as horizontal gaze palsy, were independent predictors of fall risk (43). Freezing of gait presents in 20% of patients in the first year of symptom onset and increases with disease duration (61).

Parkinsonism in RS presents as axial-predominant symmetric bradykinesia, rigidity, and less frequently tremor, usually of low amplitude postural/kinetic or mixed type (62). While at the time of diagnosis over 75 and 40% of patients with RS present symptoms of bradykinesia and axial rigidity, respectively, nearly all show these signs throughout disease course, and only 10–20% show tremor (22, 63). This is a distinguishing feature of RS from another PSP variant, PSP-Parkinsonism (PSP-P) in which tremor is present in more than half of the patients (22).

In patients with RS, dysarthria, of hypokinetic or mixed hypokinetic-spastic nature, occurs earlier and in a higher number of patients than dysphagia, although both, dysarthria and dysphagia are more frequent and more severe in RS than in other parkinsonian syndromes (64, 65). At least one third of patients have speech impairment at the time of diagnosis that leads to unintelligible speech in about 6 years and eventually involves nearly all patients (22, 66). Significant oropharyngeal dysphagia develops in about 3 to 4 years, although it might occur during the first 3 years which is an indicator of poor prognosis considering that aspiration pneumonia is the most common cause of death in these patients (67, 68). Feeding tube placement is considered after seven years of symptom onset for many patients (66).

Mild non-amnestic cognitive impairment is frequently encountered in the first 3 years and has a high risk for conversion to frontal/subcortical dementia. Older age at disease onset is the major risk factor for the development of dementia. The incidence rate of dementia in RS is estimated to be about 241 per 1,000 patients per year which is 3 times greater than that of PD (69). Cognitive changes in RS have classically been defined as a prototypic subcortical dementia with slowed central processing speed and pronounced executive dysfunction (70–72). More recent studies, separating different variants of PSP, found that phonemic verbal fluency and global cognitive performance are consistently and more severely impaired in RS (73, 74) compared to other PSP variants (75, 76). Insight is also characteristically impaired (77). Behavioral changes, mainly in the frontotemporal spectrum, are among common features of RS and in fact many patients with RS fulfill the possible bvFTD criteria (78). Social cognition, including both basic emotion recognition and more complex theory of mind functions are impaired in patients with RS. However, these patients do not usually present socially inappropriate behavior like patients with bvFTD (79). Neuropsychiatric changes are common, especially apathy and less commonly depression. Apathy affects about 80–90 % of patients with RS while depression is more common in CBS than RS (80). Sleep and eating-related disorders are probably the next common neuropsychiatric symptoms. Other behavioral symptoms with less frequency include disinhibition, aggressive behavior, irritability, and anxiety (81). In terms of impulsivity and disinhibition, a behavioral/cognitive and a motor aspect could be considered. The cognitive aspect is reflected, for example, in the Tower test. In addition to overall lower scores due to executive/planning dysfunction, patients with PSP-RS present higher number of rule-violation errors compared to other types of dementias and healthy subjects which is a marker of disinhibition. Possin et al. (82) found that, after controlling for general cognitive performance, the number of rule violation errors was correlated with the right lateral prefrontal cortex atrophy in these patients. For the motor aspect, one might think of the reckless gait, such as rocket sign and sitting en bloc as motor presentations of impulsivity. Hallucination and delusions are very rare in RS and if present are indicative of either co-pathology or medication side-effects (63, 83–85). Environmental dependency syndrome, defined as excessive dependence on environmental cues (86), which includes symptoms, such as imitation behaviors, palilalia, echolalia, echopraxia, utilization behavior, and palmar and visual grasping are also frequently encountered in patients with PSP (87, 88). Parietal lobe disinhibition due to frontal lobe and/or frontoparietal network dysfunction is believed to be the underlying cause of environmental dependency syndrome.

#### Diagnostic Criteria

The National Institute of Neurological Disorders and Stroke and Society for PSP, Inc. (NINDS-SPSP) criteria of PSP was developed in 1996, before the recognition of the majority of the clinical phenotypes of PSP pathology (45). These criteria include slowing of vertical saccades/supranuclear gaze palsy and postural instability with falls in the first year as possible and probable PSP. The NINDS-SPSP criteria have in fact high specificity for the diagnosis of the RS but moderate sensitivity (89, 90). The need for higher sensitivity and recognition of all new clinical phenotypes led to the development of the Movement Disorders Society criteria for PSP (MDS-PSP) (91) (Table 2). These criteria define three levels of certainty: suggestive, possible, and probable for each clinical phenotype to allow for the early diagnosis of patients with PSP. Wide application of the criteria revealed that the MDS-PSP criteria unexpectedly allowed for a single patient to fulfill criteria for multiple phenotypes at a time (94), which led to later development of the multiple application extinction (MAX) rules (95). Despite the new addition, which resulted in more complexity (96–98), problems remain with differentiating PSP-Parkinsonism variants from RS and to accurately diagnose PSP-Speech/language phenotype (99–102). Moreover, specificity of the criteria, especially in early stages, remains low (103).

**Table 2:** Standardized clinical diagnostic criteria of phenotypes related to primary tauopathies based on Movement Disorders Society Progressive Supranuclear Palsy (MDS-PSP) criteria, (91), Armstrong corticobasal degeneration (CBD) (24) criteria, Gorno-Tempini Primary Progressive Aphasia (PPA) criteria (92), and Rascovsky behavioral variant Frontotemporal Dementia (bvFTD) criteria (93).

	**Clinical syndrome**					
**Criteria set**	**RS**	**CBS**	**nfaPPA**	**bvFTD**	**PAGF**	**Tauopathies with Parkinsonism**
**MDS-PSP criteria** ^1^						
Probable	VSGP or SVS + Repeated falls or fall on pull test in first 3 years			VSGP or SVS + ≥ 3 of the following: • Apathy • Bradyphrenia • Dysexecutive syndrome • Reduced phonemic verbal fluency • Impulsivity, disinhibition, or perseveration	VSGP or SVS + Progressive gait freezing (Sudden, transient motor blocks/start hesitation, no/mild parkinsonism, levodopa resistant) in first 3 years	VSGP or SVS + one of: • Axial predominant, levodopa resistant bradykinesia and rigidity • Parkinsonism that is asymmetrical/with tremor/levodopa responsive
Possible	SVS + >2 steps backward on pull test in first 3 years	VSGP or SVS + Limb rigidity or akinesia or myoclonus + ≥1 cortical sign: • Orobuccal/limb apraxia • Cortical sensory deficit • Alien limb phenomena	VSGP or SVS + nfaPPA or PAOS		Progressive gait freezing in first 3 years	
Suggestive	Frequent mSWJs + Fall or >2 steps backward on pull test in first 3 years	Limb rigidity or akinesia or myoclonus + ≥1 cortical sign: • Orobuccal/limb apraxia • Cortical sensory deficit • Alien limb phenomena	nfaPPA or Progressive AOS	Frequent mSWJs or >2 steps backward on pull test in first 3 years + ≥3 of the following: • Apathy • Bradyphrenia • Dysexecutive syndrome • Reduced phonemic verbal fluency • Impulsivity, disinhibition, or perseveration		Axial predominant, levodopa resistant bradykinesia and rigidity or Parkinsonism that is asymmetrical/with tremor/levodopa responsive + one of: • Frequent mSWJs • Fall or >2 steps backward on pull test in first 3 years • s.o. PSP-SL • s.o. PSP-F • Levodopa resistant • Hypokinetic, spastic dysarthria • Dysphagia • Photophobia
**Armstrong CBD criteria**						
Probable	≥3 of: • Axial or symmetric limb rigidity or akinesia • Postural instability/falls • Urinary incontinence • Behavioral changes • VSGP/SVS	Asymmetric presentation of ≥2 cortical + ≥2 movement signs: **Cortical signs:** • Orobuccal/limb apraxia • Cortical sensory deficit • Alien limb phenomena **Movement signs:** • Limb rigidity • Limb akinesia • Limb myoclonus **Exclusionary criteria:** • Positive CSF, PET, or genetic AD biomarkers^2^ • Evidence of: LBD^3^/MSA^4^/ALS^5^/svPPA or nfaPPA • Structural lesion suggestive of focal cause • Granulin mutation or reduced plasma progranulin levels • TDP-43 mutations • FUS mutations ≥1 movement sign + ≥1 cortical sign Meeting no exclusionary criteria	Effortful, agrammatic speech + ≥1 of: • Impaired grammar/sentence comprehension with relatively preserved single word comprehension • Groping, distorted speech production (AOS)	≥2 of: • Executive dysfunction • Behavioral or personality changes • Visuospatial deficits		
**Rascovsky bvFTD criteria** ^6^						
Possible				Presence in the first 3 years of ≥3 of these symptoms: • Behavioral disinhibition^7^ • Apathy or inertia^8^ • Loss of sympathy or empathy^9^ • Perseverative, stereotyped or compulsive/ritualistic behavior^10^ • Hyperorality and dietary changes^11^ • Neuropsychological profile^12^		
Probable				All of below: • Meets criteria for possible bvFTD • Significant functional decline • Imaging results consistent with bvFTD, ≥ 1 of: • Frontal and/or anterior temporal atrophy on MRI or CT • Frontal and/or anterior temporal hypoperfusion or hypometabolism on PET or SPECT		
**Definite**				Meets criteria for possible or probable bvFTD + • Histopathological evidence of FTLD on biopsy or at post-mortem OR • Presence of a known pathogenic mutation		
**Gorno-Tempini PPA criteria** ^13^						
			**nfaPPA**	**svPPA**	**lvPPA**	
Clinical			At least one core feature: • Agrammatism • Effortful, halting speech with inconsistent speech sound errors and distortions (apraxia of speech) + ≥2 of: • Impaired comprehension of syntactically complex sentences • Spared single-word comprehension • Spared object knowledge	Both of the following core features: • Impaired confrontation naming • Impaired single-word comprehension + ≥3 of: • Impaired object knowledge, particularly for low frequency or low-familiarity items • Surface dyslexia or dysgraphia • Spared repetition • Spared speech production (grammar and motor)	Both of the following core features: • Impaired single-word retrieval in spontaneous speech and naming • Impaired repetition of sentences and phrases + ≥3 of: • Speech (phonologic) errors in spontaneous speech and naming • Spared single-word comprehension and object knowledge • Spared motor speech • Absence of frank agrammatism	
			**nfaPPA**	**svPPA**	**lvPPA**	
Imaging supported			• Clinical diagnosis of nfaPPA (as above) + ≥1 of: • Predominant left posterior fronto-insular atrophy on MRI • Predominant left posterior fronto-insular hypoperfusion or hypometabolism on SPECT or PET	• Clinical diagnosis of svPPA (as above) + ≥1 of: • Predominant anterior temporal lobe atrophy • Predominant anterior temporal hypoperfusion or hypometabolism on SPECT or PET	• Clinical diagnosis of lvPPA (as above) + ≥1 of: • Predominant left posterior perisylvian or parietal atrophy on MRI • Predominant left posterior perisylvian or parietal hypoperfusion or hypometabolism on SPECT or PET	
			**nfaPPA**	**svPPA**	**lvPPA**	
Definite			Clinical diagnosis of nfaPPA (as above) + ≥1 of: • Histopathologic evidence of a specific neurodegenerative pathology (e.g., FTLD-tau, FTLD-TDP, AD, other) • Presence of a known pathogenic mutation	Clinical diagnosis of svPPA (as above) + ≥1 of: • Histopathologic evidence of a specific neurodegenerative pathology (e.g., FTLD-tau, FTLD-TDP, AD, other) • Presence of a known pathogenic mutation	Clinical diagnosis of lvPPA (as above) + ≥1 of: • Histopathologic evidence of a specific neurodegenerative pathology (AD, FTLD-tau, FTLD-TDP, other) • Presence of a known pathogenic mutation	

AD, Alzheimer's disease; ALS, amyotrophic lateral sclerosis; AOS, apraxia of speech; bvFTD, behavioral variant frontotemporal dementia; CBD, corticobasal degeneration; CBS, corticobasal syndrome; CSF, cerebrospinal fluid; CT, computed tomography; FTLD, frontotemporal lobar degeneration; FUS, fused in sarcoma; LBD, Lewy body disease; lvPPA, logopenic variant primary progressive aphasia; MRI, magnetic resonance imaging; MSA, multiple system atrophy; mSWJs, macro-square wave jerks; nfaPPA, non-fluent agrammatic primary progressive aphasia; PAGF, progressive akinesia and gait freezing; PET, positron emission tomography; PSP, progressive supranuclear palsy; PSP-F, frontal variant of progressive supranuclear palsy; PSP-SL, speech-language variant of progressive supranuclear palsy; RS, Richardson syndrome; s.o., suggestive of; SPECT, single photon emission computed tomography; svPPA, semantic variant primary progressive aphasia; SVS, slow vertical saccades; TDP-43, transactive response DNA binding protein 43 kDa; VSGP, vertical supranuclear gaze palsy.

1 Exclusionary criteria for the MDS-PSP criteria include clinical, imaging, laboratory, and genetic markers of any PSP-mimics or differential diagnoses including AD, PD, other atypical parkinsonian disorders, motor neuron disease, vascular or other structural brain lesions, autoimmune encephalitis, metabolic encephalopathies, prion disease, sensory deficit, vestibular dysfunction, severe spasticity, lower motor neuron syndrome, leukoencephalopathy, normal pressure or obstructive hydrocephalus, Wilson's disease, Niemann-Pick disease type C, hypoparathyroidism, Neuroacanthocytosis, Neurosyphilis, Whipple's disease, MAPT, and other genetic mutations mimicking PSP clinically.

2 Laboratory findings strongly suggestive of AD such as low CSF Aβ42 to tau ratio or positive 11C–Pittsburgh compound B PET; or genetic mutation suggesting AD (e.g., presenilin, amyloid precursor protein).

3 Classic 4-Hz Parkinson disease resting tremor, excellent and sustained levodopa response, or hallucinations.

4 Dysautonomia or prominent cerebellar signs.

5 Presence of both upper and lower motor neuron signs.

6 Exclusion criteria: Pattern of deficits is better accounted for by other non-degenerative nervous system or medical disorders/Behavioral disturbance is better accounted for by a psychiatric diagnosis/Biomarkers strongly indicative of Alzheimer's disease or other neurodegenerative process.

8 At least one of: Socially inappropriate behavior/Loss of manners or decorum/Impulsive, rash, or careless actions.

8 At least one of: Apathy/Inertia.

9 At least one of: Diminished response to other people's needs and feelings/Diminished social interest, interrelatedness or personal warmth.

10 At least one of: Simple repetitive movements/Complex, compulsive or ritualistic behaviors/Stereotypy of speech.

11 At least one of: Altered food preferences/Binge eating, increased consumption of alcohol or cigarettes/Oral exploration or consumption of inedible objects.

12 All of: Deficits in executive tasks/Relative sparing of episodic memory/Relative sparing of visuospatial skills.

13 Inclusion criteria: most prominent clinical feature is difficulty with language; these deficits are the principal cause of impaired daily living activities; aphasia should be the most prominent deficit at symptom onset and for the initial phases of the disease. Exclusion criteria: none of these criteria apply: pattern of deficits is better accounted for by other non-degenerative nervous system or medical disorders; cognitive disturbance is better accounted for by a psychiatric diagnosis; prominent initial episodic memory, visual memory, and visuoperceptual impairments; prominent, initial behavioral disturbance.

#### Differential Diagnosis

Corticobasal degeneration-Richardson syndrome (CBD-RS) presents with a similar clinical picture as described above and clinical distinction from PSP-RS is sometimes very challenging. Few studies compared clinical features of the two tauopathies. Behavioral abnormalities and urinary incontinence were significantly associated with CBD-RS compared to PSP-RS in one autopsy-confirmed study (104). Among early features, dysarthria was more frequent in PSP-RS while memory complaints were more common in patients with CBD-RS. It is suggested that the presence of CBS-specific clinical features (such as increased saccadic latency, cortical sensory loss, action myoclonus, limb or orobuccal apraxia, and limb dystonia or rigidity, especially in an asymmetric pattern) in a patient with RS is probably indicative of an underlying CBD pathology, but these features may occur relatively late (105, 106). Integration of biomarkers into current diagnostic criteria might be a necessary next step to improve the diagnostic accuracy. We will discuss recent advancements in the field of biomarkers in a later section.

### Corticobasal Syndrome

In 1967, Rebeiz et al. (107) described three patients with progressive myoclonus, dystonia, gaze limitation, akinesia and monolimbic rigidity, speech disorder, and limb apraxia. Frontoparietal atrophy with special type of neuronal achromasia was evident in pathologic examination. It was initially called “corticodentatonigral degeneration with neuronal achromasia.” Since cerebellar features were not part of the clinical syndrome, its terminology changed to corticobasal ganglionic degeneration and later to CBD (108). Presently, CBD is considered a pathologic entity with hyperphosphorylated 4R-tau accumulation in astrocytes forming the astrocytic plaques which is the hallmark of pathology (109). A constellation of symptoms originally thought to be specific for CBD pathology is currently called corticobasal syndrome (CBS). Incidence of CBS is estimated to be 0.4 per 100,000 (49) which is less common than RS as an atypical parkinsonism. Mean age at disease onset is about 64 years. It seems to be a little more common in females and has a mean survival of about 6.5 years (24).

#### Clinical Characteristics

Corticobasal syndrome symptoms include orobuccal and/or limb apraxia, cortical sensory loss, alien limb phenomena, asymmetrical limb rigidity/akinesia, limb myoclonus, and limb dystonia. The first three symptoms are classified as cortical and the other three as motor symptoms.

Although parkinsonism is typically asymmetric (24), there are reports of symmetric parkinsonism (110). A minority of parkinsonian syndromes in CBS is levodopa responsive. Tremor is not common and is usually postural or action induced with a jerky myoclonic-like quality (24). About 40% of patients suffer from dystonia which is usually asymmetric and is more common in upper limbs. Lower limb dystonia, blepharospasm, and cervical dystonia is not common in CBS. Dystonia is usually associated with myoclonus and presents during the first 2 years of disease onset (109). Myoclonus is usually of the cortical reflex type (111) and has been proposed to be the result of lack of inhibitory input from the sensory cortex (112).

Apraxia is the clinical hallmark of CBS. Although it was believed that apraxia is also specific to CBS, it can be seen in other parkinsonian syndromes especially RS but is not as severe (113, 114). The alien-limb syndrome usually associates with apraxia, but these symptoms are unrelated (115). An alien limb is associated with a sense that the limb is foreign and acts on its own, which is not characteristic of apraxia (115). There are two alien-limb syndromes. In the sensory or posterior type, the patient believes that the limb is not their own and levitation may be present (116). In the anterior or motor type, the patient shows utilization-dependent limb activities which may result in inter-manual conflict (117).

#### Diagnostic Criteria

Based on the most recent clinical CBD criteria by Armstrong et al. (24), asymmetric presentation of a combination of at least two of these cortical and at least two of these motor symptoms leads to the diagnosis of probable CBD-CBS while the presence of one cortical plus one motor symptom suggests the diagnosis of possible CBD-CBS. It is important to note that exclusion criteria exist for both categories and include the evidence of biomarkers to exclude Alzheimer's disease [AD; such as amyloid positron emission tomography (PET) or tau and amyloid in cerebrospinal fluid (CSF)], structural lesion suggestive of focal causes, granulin mutation or reduced plasma progranulin levels, TDP-43 mutations, fused in sarcoma (FUS) mutations, evidence of Lewy body disease (LBD), multiple system atrophy (MSA), amyotrophic lateral sclerosis, or svPPA (24).

#### Differential Diagnosis

Although CBS is the most common clinical phenotype of CBD (37%), there are other clinical phenotypes that CBD can present with, including RS (23%), frontal behavioral-spatial (13%), amnestic syndrome of the hippocampal type (8%), nfaPPA (5%), and other rare presentations including PD-like or mixed phenotypes (Figure 1) (24). On the other hand, CBS phenotype may be the predominant clinical picture of various pathologies other than CBD in addition to PSP (PSP-CBS), including PiD (PiD-CBS), Alzheimer's disease pathology (AD-CBS), transactive response DNA binding protein 43 kDa (TDP-43) (TDP-CBS), and rarely LBD, GGT, vascular pathology, or even sporadic Creutzfeldt- Jacob disease (sCJD) (25, 32, 118–121).

There are few studies comparing CBD-CBS and PSP-CBS. In a small clinicopathologic study of 11 cases, eyelid opening apraxia, early falls, and predominant downgaze abnormalities were more frequent in patients with PSP-CBS while cortical sensory loss and limb clumsiness or alien limb were more common in CBD-CBS (106).

A number of studies compared AD-CBS to CBD-CBS (122–129) and found that in AD-CBS myoclonus, memory impairment, dressing apraxia, visuospatial neglect, and hallucinations were more common while in CBD-CBS dystonia, falls, tremor, and early frontal behavioral symptoms were more frequent and MMSE scores were higher at the disease onset. Boyd et al. (129), in an interesting pathology-confirmed study, found that the spatial subset of a visuospatial battery, more specifically the cube analysis test, was significantly impaired in patients with AD-CBS compared to CBD-CBS. This finding is consistent with the preferential involvement of the dorsal stream in parietal lobes by Alzheimer's disease (AD) pathology in AD-CBS cases.

TDP-43 proteinopathy Mackenzie type A [Sampathu type 3 (130)] may present with CBS as one of its clinical phenotypes which also include bvFTD with or without motor neuron disease (MND) and nfaPPA (26, 131, 132). In TDP-CBS, the related underlying genetic predisposition, if present, is one of the progranulin gene variants (133, 134). There are no reports comparing the clinical features of CBS with underlying TDP-43, LBD, or GGT to CBD-CBS. Vascular pathology and sCJD-CBS could be diagnosed based on the relevant MRI findings and a very rapid course of less than 12 months in case of sCJD (118, 120).

It is very challenging to determine the underlying pathology of CBS solely based on clinical grounds. However, if the Armstrong CBD exclusionary criteria that includes the use of biomarkers to exclude AD (using CSF or PET biomarkers), TDP-43, or FUS are applied, the set of diagnostic criteria will be very specific. Unfortunately, attempts to validate the diagnostic criteria of CBD-CBS failed to do so because none of the investigators used biomarkers as exclusionary criteria (135, 136). This again underlines the importance of integrating biomarkers to the clinical criteria.

### Non-fluent/Agrammatic Primary Progressive Aphasia

The non-fluent agrammatic variant of primary progressive aphasia (nfaPPA) is very specific for an underlying tauopathy. CBD is the most common tau pathology (44%) followed by PSP (24%), and PiD (16%). Non-tauopathy nfaPPA is usually associated with TDP-43 type A or AD pathologies (Figure 1) (27). A minority of nfaPPA cases have a genetic etiology, commonly in association with either GRN or C9orf72 but only rarely with the MAPT gene (137–139). Incidence of nfaPPA is estimated about 0.9 per 100,000 per year based on a recent population-based Italian study (140). NfaPPA is usually accompanied with apraxia of speech, in more than half of the cases, although, the two syndromes may occur in isolation (141, 142). Based on the current clinical criteria, the two disorders are merged under the term nfaPPA. However, it is now understood that the two disorders are separate entities although they often co-occur.

#### Clinical Characteristics

Clinical hallmark of nfaPPA is agrammatism and effortful, distorted speech. Agrammatism is a a primary disorder of the language system and the term, primary agrammatic aphasia is used when it occurs as an isolated neurodegenerative process (143). Agrammatism is primarily a disorder of language and symptoms include moderate to severe decrease in the fluency or verbal output, a telegraphic speech with frequent loss of articles and conjunctions. As a part of agrammatism, understanding of syntactically complex sentences is particularly impaired. Early in the disease course, writing might be spared (144). Apraxia of speech is essentially a motor speech disorder in which motor programs necessary for the production of phonetically and prosodically normal speech are impaired (145). A pure, progressive, neurodegenerative apraxia of speech without involvement of the language system is then called primary progressive apraxia of speech. Symptoms include mispronunciation of words, speech sound distortion, visible or audible groping to find the suitable movements to articulate, false start, restart, omission, or addition of distorted sounds, all in an effortful and deliberately slow rate. Milder degrees of dysarthria may accompany both agrammatism and apraxia of speech (143, 146). Since agrammatism and apraxia of speech both affect speech output and effortful, nonfluent speech is a feature of both disorders, differentiation between agrammatism and apraxia of speech might be challenging. However, in contrast to agrammatism, speech sound production errors and sound distortion is seen in patients with apraxia of speech. In addition, syntax and comprehension of syntactically complex sentences is preserved in isolated forms.

#### Diagnostic Criteria

Either agrammatism or apraxia of speech could be the core criteria which defines probable nfaPPA in combination with at least two of these three symptoms: impaired comprehension of syntactically complex sentences, spared object knowledge, and spared single-word comprehension (Table 2) (92). The current PPA criteria have been challenged for being non-specific in differentiating between logopenic and nfaPPA variants. There are clinical suggestions for the improvement of its specificity and several new biomarkers can also be helpful to differentiate AD and logopenic variant from patients with a nfaPPA tauopathy (147).

#### Differential Diagnosis

Although by definition, speech/language features should be the most prominent deficit on symptom onset and in the first few years, other clinical symptoms usually accompany nfaPPA/apraxia of speech after 2–3 years of disease onset. Especially, those with an underlying PSP (PSP-SL) or CBD (CBD-nfaPPA) pathology will probably develop parkinsonism, gait disturbance, limb apraxia, dystonia, and sometimes vertical supranuclear gaze palsy/slow vertical saccades. Other features that might predict an underlying tauopathy, far more commonly a 4R tauopathy, include apraxia of speech as the first presentation, higher general cognitive performance, presence of behavioral impairment, and early occurrence of mixed/hypokinetic dysarthria (27, 148). Early motor speech disorder has specifically been linked to a PSP pathology (149). A pathology-based study showed that among patients fulfilling the criteria for PSP-SL, those with probable PSP-SL are more likely to have an underlying PSP pathology, while those with suggestive-of-PSP-SL are more consistently associated with a CBD pathology (101). In a recent study, survival was about 6 years for patients presenting with primary progressive apraxia of speech, around 5 years for agrammatic aphasia, and about 4 years for combined apraxia of speech and agrammatism (150).

### Frontal Behavioral/Dysexecutive Syndrome

About 30% of patients presenting with bvFTD suffer from some form of tauopathy, almost equally distributed between PiD, CBD, and PSP. More than 50% are due to TDP-43 proteinopathies and about 13% have an AD pathology (Figure 1) (35). The typical clinical picture of bvFTD, which is dominated by PiD, TDP-43, and FUS pathologies, includes disinhibition, apathy, loss of sympathy/empathy, perseveration or compulsive/ritualistic behavior, hyperorality, and executive dysfunction with relatively spared episodic memory and visuospatial domain (93). The presence of at least 3 of the above 6 features in addition to frontal/anterior temporal atrophy or hypometabolism on brain imaging in a setting of progressive functional decline defines probable bvFTD (93).

In a large pathology-based study (35), language dysfunction, depression, and myoclonus were more common in patients presenting with a frontotemporal syndrome with an underlying AD compared to all other pathologies while falls, dysphagia, dysarthria, mental rigidity, aggression, apathy, and lack of insight, were against an AD pathology. Recently, the term behavioral variant AD (bvAD) was introduced by Ossenkoppele et al. (151) in their systematic review and meta-analysis of bvAD vs. bvFTD. Ossenkoppele et al. found that behavioral profile is generally milder in bvAD; the patients have fewer compulsive behaviors and less hyperorality but more agitation, delusions, and hallucinations, and more severe memory and/or executive dysfunction compared to bvFTD. These findings were incorporated in the research criteria for bvAD (151). Fronto-behavioral AD, frontal variant AD, and frontal/dysexecutive AD are other terms that have been used to describe the population of patients with a frontal-dominant dementia syndrome and an underlying AD pathology.

It has been suggested that accompanying symptoms of MND or semantic dementia are indicative of TDP-43 pathology, type B and C, respectively (26, 36). Moreover, executive dysfunction occurred earlier and was more severe in fronto-behavioral AD cases (35, 152). Features against an underlying tauopathy include earlier age of onset, delusions, visual misperceptions, and signs of MND. In contrast, binge eating was in favor of bvFTD tauopathy (35, 153). Loss of empathy and compulsive behavior were less common in frontal variant PSP (PSP-F) or CBD-frontal behavioral-spatial syndrome (CBD-FBS) compared to PiD or bvFTD with non-tauopathies but dysarthria and dysphagia were more frequent in PSP-F (35). In another study, patients with fronto-behavioral AD were less likely to have personality changes but frequently showed memory impairment. Personality change was significantly higher in tauopathies presenting with bvFTD compared to non-tauopathies (154). The MDS-PSP criteria for PSP-F requires at least three of the following symptoms: apathy, bradyphrenia, dysexecutive syndrome, reduced phonemic verbal fluency and impulsivity, disinhibition, or perseveration, in addition to vertical supranuclear gaze palsy/slow vertical saccades (91). However, vertical supranuclear gaze palsy/slow vertical saccades occurs in <20% of patients during the first 4 years of symptom onset which increases diagnostic latency in cortical variants of PSP (21, 155). Misdiagnosis as psychiatric disorders is another common source of diagnostic delay in patients presenting with bvFTD syndrome (156).

Globular glial tauopathy presents with bvFTD syndrome in 39 and 27% of type I and III cases, respectively (34). Most common genetic causes of bvFTD syndrome include C9orf72 repeat expansions and GRN and MAPT mutations. MAPT mutations cause tauopathies that present in about 45% of cases with a bvFTD syndrome followed by unspecified dementia (34.6%), parkinsonian syndrome (4.9%), RS (4.2%), amnestic syndrome (3%), CBS (1.8%), nfaPPA (1.8%), and svPPA (1.8%) phenotypes (157). GRN mutations and C9orf72 repeat expansions present with the similar range of clinical syndromes; however, PPA syndromes are more common with GRN mutations and motor neuron disease syndromes are more common with C9orf72 repeat expansions (157). Pro301Leu MAPT mutation is the most common cause of genetic tauopathy followed by IVS10 + 16C>T, Arg406Trp, and Asn279Lys. Parkinsonian symptoms, sometimes mimicking RS and/or CBS, are common later in the course of MAPT mutations presenting with bvFTD or other syndromes (158). Although penetrance is almost 100%, age of onset is highly variable ranging from the first decade to 82 years (157) and there is both inter- and intra-familial variability in clinical presentation and symptoms.

### Progressive Akinesia and Gait Freezing (PAGF)

Progressive supranuclear palsy-progressive akinesia and gait freezing (PSP-PAGF) is the only tauopathy presenting with this clinical syndrome. It was first reported in 1980 from Japan with subsequent pathologic confirmation of underlying PSP (159, 160). The clinical picture is dominated by the freezing of gait with frequent start hesitation and blocking, but freezing could also involve other movements, such as speech and writing (161). Axial rigidity might be present; however, typical parkinsonian features, such as limb rigidity or bradykinesia and tremor are lacking, and symptoms do not respond to levodopa. Frequent falls follow severe gait freezing. Speech becomes hypophonic and writing is rapid micrographic. Hypomimia is also a frequently reported symptom (161–163). Typical PSP features, such as impaired postural reflexes, vertical supranuclear gaze palsy/slow vertical saccades, eyelid opening apraxia, dysarthria, dysphagia, apathy, and cognitive frontal impairment are delayed by at least 5 years and usually 8–10 years (161, 162, 164). Some patients may never develop vertical supranuclear gaze palsy/slow vertical saccades or dementia during life (163, 165). Other etiologies, such as vascular white matter disease (166) and carbon monoxide poisoning (167) have been ascribed to PAGF syndrome. However, their courses are different from neurodegenerative diseases and other clinical features, especially more apparent parkinsonism and pyramidal signs, differentiate these disorders from PSP-PAGF. A case of PAGF with subsequent progression to a corticobasal syndrome has also been reported but pathologic diagnosis was lacking in this report (168).

## Clinical Syndromes With Less Association to a Primary Tauopathy

### Parkinsonian Syndrome

Progressive supranuclear palsy-Parkinsonism (PSP-P) is the second most common PSP phenotype, accounting for about 37% of PSP cases (20, 22). PD is the most common alternative diagnosis when a patient with PSP first approaches a neurologist (20). PSP-P is the most common tauopathy that closely resembles PD, presenting with asymmetric akineto-rigid parkinsonism, rest tremor, and relatively good levodopa response (169). Williams et al. (22) first introduced this phenotype. The mean age of the onset of PSP-P was similar to PSP-RS in Williams' series. However, other studies reported higher age of onset of about 68 years (21). This phenotype is considered as one of the brainstem restricted forms of PSP with survival of about 9–13 years (22, 169, 170), longer than RS but shorter than PD, so it is crucial to differentiate the three disorders. It is important to emphasize that even at the same baseline disease severity, patients with PSP-P progress at a slower pace than patients with RS (171). It is, however, difficult to diagnose PSP-P early in the disease course and diagnosis is often delayed by up to 10 years of symptom onset because characteristic PSP features, vertical supranuclear gaze palsy, and postural instability, occur relatively late in patients with PSP-P (21).

Postural instability usually does not occur in the first 2–3 years of symptom onset in PSP-P and vertical supranuclear gaze palsy/slow vertical saccades has a latency of about 5 years in these patients while parkinsonism occurs in the first year (99, 169). In about 40–80% of patients, parkinsonism starts asymmetrically, 50% of patients present with tremor, and about 50–70% of patients have moderate to good response to levodopa early in the disease course (169, 172). While some studies found no difference in apathy or depression between PSP-P and RS (76, 173), apathy was more frequent in PSP-P in one study (75) and in PSP-R in another (174), but further studies are needed. Early cognitive decline is more common in RS than PSP-P (76, 172) and generally, neuropsychiatric and cognitive symptoms are more severe in both PSP variants compared to PD (76). On the other hand, PD-specific features, such as autonomic dysfunction and levodopa-induced dyskinesia are not usually seen in patients with PSP-P (169).

### Late Onset Cerebellar Ataxia Syndrome (LOCA)

A cerebellar ataxic syndrome is very unspecific for tau pathology since a lot of genetic, autoimmune, degenerative, and many other structural disorders are present with this syndrome. PSP-Cerebellar (PSP-C) is the only rare tauopathy that presents with a cerebellar syndrome. It was first proposed as a PSP phenotype when retrospective evaluation of a Japanese autopsy series with pathological diagnosis of PSP revealed that some cases had a primary and predominant syndrome of cerebellar ataxia before the presence of PSP-specific features. There was abundant tau pathology involving Purkinje cells and more severe tau pathology in dentate nucleus (175). Prospective autopsy-confirmed cases were reported thereafter (176, 177). Some of these patients had been diagnosed with a form of spinocerebellar ataxia. The closest neurodegenerative differential diagnosis is multiple system atrophy-cerebellar (MSA-C) which could be differentiated because their relatively younger age of onset, early onset of more severe autonomic disturbances, lower rates of falls, and vertical supranuclear gaze palsy/slow vertical saccades, present hot-cross-bun sign on brain MRI (178–180). In recent reviews of 16 PSP-C cases, of which 12 were pathology-proven, the mean age of onset was 65 (range 57–73) years, mean disease duration was about 6 years, and symptoms were mostly restricted to a cerebellar ataxia syndrome for about 2 years (181). Interestingly, some patients never developed vertical supranuclear gaze palsy.

### Primary Lateral Sclerosis

A few patients presented have been reported with pyramidal symptoms including spastic gait and speech indicative of primary lateral sclerosis (PLS) but the pathological diagnosis was PSP in the absence of any PLS-related pathologies, such as TDP-43 or FUS proteinopathies (182, 183). These findings suggest that PSP-PLS might be a rare PSP phenotype.

Prior to these reports, a retrospective study of 12 autopsied cases with prominent upper motor neuron as well as extrapyramidal symptoms revealed atypical PSP-like pathological changes, with Gallyas-negative tufted astrocytes and minimal involvement of subcortical nuclei typically involved with PSP, but with significant degeneration of the corticospinal tract due to 4R tau pathology (184). Subsequent studies led to further characterization of the distinct pathological changes and identified them as a new pathological entity named globular glial tauopathy (GGT) (7, 185). The GGT has been divided pathologically into three subtypes: type I, involving frontotemporal areas and causing frontotemporal dementia (FTD); type II, which involves motor cortex and the descending corticospinal tracts causing MND; and type III that is a combination of the first two (FTD-MND). In all types, extrapyramidal symptoms might be a part of the clinical picture (7). Thus, PLS syndrome is associated with GGT types II or III and mostly in combination with other symptoms and sometimes other pathologies (34, 42). Recent cryo-EM studies suggest that GGT type III might be a distinct pathological entity since its tau filament structure does not twist, unlike the other types (16).

### Other Primary Progressive Aphasias

Semantic variant PPA (svPPA) is a clinical syndrome tightly associated with an underlying TDP-43 (mostly type C) pathology. However, <15% of patients may have an underlying tauopathy of PiD or GGT type and about similar proportion are due to AD pathology (27, 186–188). Bilateral involvement of the anteromedial and inferior temporal areas more severely on the dominant side could be appreciated on the structural or functional neuroimaging of svPPA (189). Based on the most current clinical criteria, the core clinical features required for the diagnosis of svPPA include deficits in both confrontation naming and single word comprehension as a result of progressive defect in semantic memory (92). Motor aspects of speech, fluency, and grammar are characteristically spared, and patient might even be verbose; however, the speech content lacks meaning. More generic category names might be used instead of specific names and filler words are used more frequently. Surface dyslexia and dysgraphia might also be present. Behavioral features are not uncommon in svPPA; however, they are more commonly encountered in the right-sided semantic dementia. Extrapyramidal motor features when present in the course of svPPA are consistent with an underlying tau rather than TDP pathology (27) while a temporoparietal pattern of brain atrophy on imaging are probably indicative of underlying AD (190).

Logopenic variant PPA (lvPPA) is believed to be associated with an underlying AD pathology in most of the cases (27, 191, 192). Non-AD pathologies presenting with lvPPA include TDP-43 type A, LBD, CJD, and rarely tauopathies, such as CBD, PSP, and PiD (141, 188). Core clinical features of lvPPA include impairment in single-word retrieval and repetition of sentences and phrases which are probably due to a malfunctioning phonological loop of verbal working memory (192). Recently, Mesulam et al. (147) argued that the current clinical criteria for lvPPA are unspecific and allow for misclassification of other variants, especially nfaPPA, into logopenic category. Considering that the rate of amyloid beta biomarker positivity is the highest in lvPPA compared to other PPAs; evaluation of these biomarkers might be useful in differentiating the two variants (188).

### Amnestic Syndrome of the Hippocampal Type

Alzheimer's disease is the most common secondary tauopathy. As a mixed or pure pathology, it is the major cause of an amnestic syndrome in about 56–90% of cases (28–30). Other pathologies that may present with amnestic syndrome include limbic-predominant age-related TDP-43 encephalopathy (LATE), non-AD tauopathies, LBD, vascular, CJD, and mixed pathologies (29). Non-AD tauopathy categories presenting with amnestic syndrome include AGD, primary age-related tauopathy (PART), CBD, GGT, and PiD.

Argyrophilic grain disease, a 4R tauopathy first introduced in 1987 (10), has been associated with many psychiatric symptoms including emotional instability, delusions, hallucinations, depression, anxiety, and personality changes (193, 194). Although an Alzheimer-like dementia seems to be the dominant clinical presentation of this age-related tauopathy, in a large pathological study, about 59% of the cases with an AGD pathology had no cognitive impairment (38). AGD has a prevalence of 5–15% in community-based autopsy studies and usually co-occurs with other neurodegenerative disorders, especially AD and other age-related pathologies including PART and hippocampal sclerosis (38, 195). Therefore, it is challenging to attribute cognitive decline to AGD alone but preferential involvement of the limbic and hippocampal structures in AGD supports its contribution to cognitive decline (195, 196).

Primary age-related tauopathy is a mixed 3R/4R tauopathy commonly found in the brains of cognitively normal individuals in the form of neurofibrillary tangles restricted to the medial temporal lobes and structures typically involved in AD but with only minimal neocortical involvement. Amyloid beta plaques are either absent (definite PART) or mild (possible PART) (15). When symptomatic, PART usually presents with a slowly progressive cognitive decline which starts at a higher age and progresses at a slower pace compared to AD, and when associated with AD pathology, hastens cognitive decline (39, 40).

Pick's disease constitutes the postmortem diagnosis of a minority of amnestic syndrome cases in autopsy series, ranging from 4 to 10% (28, 31). Early occurrence of frontal behavioral symptoms have been reported in these patients as a distinguishing clinical feature (197).

Compared to typical AD, CBD-amnestic patients are younger and several features including hyperreflexia, gait abnormalities, parkinsonism, dystonia, and asymmetric motor/sensory symptoms are significantly more frequent in these patients early in the disease course. Falls, urinary incontinence, and ocular movement abnormalities are distinguishing clinical features in later stages (198).

Memory impairment is the predominant clinical syndrome in <10% of patients with GGT (34). AD-mimicking presentation has rarely been reported in association with this tauopathy (199).

### Asymptomatic Tauopathies

Aging-related tau astrogliopathy (ARTAG) is an essentially asymptomatic 4R tauopathy that exclusively involves astrocytes in various cortical and subcortical brain areas in individuals older than 60 years (11). ARTAG is frequently encountered as a single pathology in individuals with no neurologic symptoms or as a co-pathology along with many neurodegenerative disorders (11). In a large community-based autopsy series, 38% of brains over 75 years of age contained cortical ARTAG (200). Although, parkinsonism, aphasia, or cognitive decline have been reported in association with this tauopathy, clinical significance of ARTAG is yet to be determined (201, 202).

Incidental finding of mild sub-threshold stages of many tauopathies have been reported in autopsy series. Incidental PSP has been reported in 2.5–6.9% of community-based autopsy series (203–205) and the rate of incidental CBD was about 0.23% in a Japanese forensic autopsy series (206). AGD and PART, as stated above, usually present as asymptomatic tauopathies in autopsy studies.

## Biomarkers to Differentiate Tauopathies Sharing a Clinical Phenotype

Accurate *in vivo* diagnosis of the underlying pathology is yet an unsolved issue facing all therapeutic clinical trials of tauopathies. Clinical diagnostic criteria are currently used both in clinical and research settings as the basis for diagnosing various tauopathies from one another and from non-tauopathy disorders. However, clinical heterogeneity and huge phenotypic overlap of different tauopathies, as discussed above, necessitates the development and inclusion of biomarkers into the clinical diagnostic criteria of tauopathies in the near future to improve their diagnostic accuracy, as previously tested in diagnostic criteria of AD (207, 208).

In the field of diagnosis, biomarkers could be helpful in (1) the early diagnosis of the clinical phenotype when the clinical picture of a tauopathy is not yet complete to fit in a specific clinical criteria; (2) confirming a syndromic diagnosis when clinical findings are conflicting or a patient fulfills the criteria for multiple phenotypes; (3) molecular diagnosis of the underlying etiology/proteinopathy; and (4) as exclusionary criteria.

Structural MRI measures can supplement clinical findings to confirm a given phenotypic diagnosis or possibly can be used for the early detection of clinical syndromes. However, considering the scarcity of evidence from pathology-confirmed studies, not only the correlation of specific conventional or quantitative MRI measures with specific pathologic diagnoses is not yet known, but structural imaging cannot differentiate the underlying pathology. PET and fluid biomarkers are rapidly evolving to address this purpose. On the other hand, frequent presence of associated pathologies is a major hinder that prevents molecular diagnoses to rest on one biomarker alone. Therefore, a combination of various biomarkers might be necessary for more accurate diagnosis.

### Structural MRI

Midbrain and superior cerebellar peduncle atrophy and their derivatives, such as magnetic resonance parkinsonian index (MRPI) and MRPI 2.0, are hallmark MRI findings of RS which can distinguish this clinical syndrome from healthy controls, other atypical parkinsonian syndromes, and PD (209–211). However, these indices cannot differentiate between RS due to various pathologies (e.g., PSP-RS from CBD-RS) (212).

In a longitudinal study, all patients with a clinical diagnosis of PSP-P and an abnormal MRPI developed vertical supranuclear gaze palsy during 4 years of follow up (213). In another study, 73% of patients with undifferentiated parkinsonism who had abnormal MRPI fulfilled possible or probable PSP criteria up to 4 years after MRI acquisition (214). A recent study evaluated brainstem atrophy measures including midbrain area, midbrain/pons ratio, and MRPI across eight PSP variants who were clinically diagnosed using the MDS-PSP criteria. Results showed no difference between brainstem and cortical variants, but of note, possible PSP-SL patients had less midbrain atrophy while PSP-CBS and PSP-F patients had highly abnormal indices. Among 26% of the cases in this study who underwent an autopsy, one third had non-PSP pathology dominated by SL phenotype (215). These studies, although mostly lacking pathologic confirmation, show that midbrain/superior cerebellar peduncle atrophy and MRPI might be useful in the early diagnosis of RS or other probable/possible variants by improving the sensitivity of the clinical criteria in the first visit.

In a conventional MRI prominent atrophy, peri-rolandic cortices in an asymmetric pattern was thought to be specific to CBS (216, 217), although this is again not specific to any underlying pathology (218, 219). Voxel-based morphometry (VBM) studies confirmed these findings and showed atrophy involving primary and supplementary motor and parietal, especially superior parietal lobule, cortical areas prominently in the side contralateral to the symptomatic limb; these changes are probably helpful in differentiating CBS from other parkinsonian phenotypes especially PD, MSA-Parkinsonism, and RS (220–223). In a comparative VBM MRI study of CBS due to four common pathologies of CBD, PSP, AD, and TDP-43, Whitwell et al. (224) found that more widespread cortical atrophy is indicative of an underlying AD or TDP-43 while more focal atrophy of premotor and supplemental motor area is seen with CBD and PSP .

In nfaPPA, due to tauopathies, MRI atrophy was found on the left posterior frontal cortex especially the opercular part of the inferior frontal gyrus, premotor and supplementary motor areas, and striatum along with severe frontal white matter atrophy, while in nfaPPA, due to TDP-43, white matter was spared (148). More specifically, the proportion of gray matter to white matter atrophy was found to be higher in CBD-nfaPPA compared to PSP-SL which showed more severe contraction of the white matter running between the striatum, premotor, and prefrontal areas (149). More focal involvement of the motor, premotor, and supplementary motor areas have been reported in the primary progressive apraxia of speech (225).

Patients with bvFTD irrespective of the underlying pathology share atrophy in the anterior cingulate, fronto-insula, striatum, and amygdala areas (35). PiD-bvFTD shows more widespread atrophy of the right frontal and anterior temporal lobes while CBD and PSP had less anterior temporal atrophy (35). Marked atrophy of bilateral temporoparietal regions with minimal frontal atrophy is indicative of an underlying AD (152). Parietal atrophy is also more severe in TDP-43 pathology compared to PiD or CBD (226), and the accompanying midbrain atrophy is indicative of an underlying PSP (227).

A recent study evaluated the pattern of covarying brain atrophy and clinical features across a mixed sample of clinically diagnosed patients including RS, CBS, bvFTD, and PPA variants. It showed that these syndromes exist as a continuous spectrum of structural imaging and clinical features, in the three dimensions of behavior, movement, and language, rather than discrete entities. Overlap is common and increases with time (228).

### Positron Emission Tomography

Tau PET tracers are viewed as the future of *in vivo* pathological diagnosis of tauopathies. Flortaucipir ([18F]AV- 1451), was the first of its kind to be approved by the FDA on May 2020 for *in vivo* diagnosis of tau pathology in AD (229). Despite having high sensitivity and specificity for higher stages of AD pathology (230), flortaucipir showed much lower, but still significant, affinity to non-AD tau fibrils and, in this regard, significant off-target binding that is seen with this tracer (231) is a greater challenge in non-AD tau imaging. Although flortaucipir binds with reduced affinity to pathologic tau in non-AD tauopathies, such as PSP and CBD (232–234), it is not specific to a single type of tau fibril (235). Nevertheless, tracer uptake in probable CBD-CBS was distinct compared to AD-CBS which showed more diffuse uptake especially in the temporal and parietal areas (234, 236). These findings can be used in the differentiation of the underlying pathology in CBS. In a more recent study on patients with PSP of various clinical phenotypes, Whitwell et al. (19) found that flortaucipir uptake was correlated with areas of expected involvement and MRI volume loss and in some autopsied cases with postmortem tau burden. In this study, putamen and globus pallidus uptake was the highest with PAGF and parkinsonism variants, and supplementary motor and precentral cortices with speech/language variant.

An earlier first generation tracer, [11C]PBB3 and its derivatives, [18F]PBB3 and [18F]-PM-PBB3 ([18F]APN-1607), are also among tracers with acceptable non-AD tau binding affinity with some off-target binding in the basal ganglia, thalamus, and choroid plexus but not to monoamine oxidase (MAO) (237). In a recent study comparing clinically diagnosed patients with PSP to healthy controls, Endo et al. (238) found that [11C]PBB3 binding to frontoparietal white matter is correlated with disease severity in patients with PSP. Still another study showed differential [11C]PBB3 binding, reflecting areas typically involved with tau pathology, in clinically diagnosed PSP, CBS, and AD cases (239). However, significant [11C]PBB3 binding to alpha-synuclein and TDP-43 has been reported in other studies (240, 241).

The newer [18F] APN-1607 was recently tested in patients clinically diagnosed with PSP and MSA and the results showed that tracer uptake in the brainstem structures including the subthalamic nucleus, midbrain, substantia nigra, red nucleus, pontine base, and raphe nuclei was associated with disease severity in PSP. Moreover, the basal ganglia and the midbrain uptake could differentiate PSP from MSA (242). Trials are ongoing for the evaluation of [18F] APN-1607 in a larger sample of various parkinsonian disorders including PSP and CBD (NCT04557865, ClinicalTrials.gov).

Many second generation tau tracers have improved the affinity and reduced the off-target binding but remained mainly AD-specific (237). More recently, [18F]-PI-2620 was introduced as a new second generation tau tracer with better affinity to PSP, CBD, and PiD but still with relatively much higher affinity for AD (243). Clinical studies, however, were not able to find a consistent binding to non-AD tau. Moreover, there was no postmortem correlation to tau pathology in a CBD case (244). However, in another study using a dynamic technique, [18F]-PI-2620 was able to differentiate PSP-RS from healthy controls, PD, MSA, and non-RS PSP (245) and in a follow-up study, an improved imaging technique was proposed (246). Palleis et al. (247) compared patients with CBS having underlying AD, based on amyloid-PET imaging, and non-AD tau and suggested that higher dorsolateral prefrontal cortical binding of this tracer may differentiate AD-CBS from tau-CBS. None of the first or second-generation tau tracers is able to differentiate between various 3R or 4R tauopathies.

Cryo-EM ultrastructural evaluation of tau fibrils studies show that among tauopathies, CBD, AGD, and ARTAG fibrils have more structural resemblance (four-layered fold), while PSP fibrils are similar to those of GGT (three-layered fold), and PART and AD fibrils are identical (16). This suggests that different tau tracers might be needed for the diagnosis of CBD and PSP. A new 4R-specific tau tracer, [18F]CBD-2115, has been developed by Lindberg et al. on 2021 which showed higher affinity for PSP-specific 4R tau compared to flortaucipir in preclinical evaluations (248). Clinical studies are yet to be done.

### Cerebrospinal Fluid and Serum Biomarkers

Cerebrospinal fluid levels of total tau, phosphorylated tau 181 (p-tau181), and amyloid beta 1-42 (Aβ42) have been studied for more than 30 years for the diagnosis of AD pathology (249), especially as an early diagnostic tool and eventually incorporated into the diagnostic criteria of AD in 2018 (207). Wagshal et al. (250) showed that CSF p-tau181 and total tau are decreased in RS (250) in sharp contrast to what occurs in AD. In another study, Lleo et al. (251) showed that FTLD-tau generally have lower total and p-tau levels compared to AD, but patients with sporadic FTLD-TDP had even lower p-tau181 compared to PSP for which they could be used as a diagnostic marker. This was confirmed by a recent study using pathologically-confirmed cases and automated laboratory techniques (252). These investigators found in this relatively large sample of AD and FTLD cases including tauopathies and TDP-43opathies that CSF Aβ42/Aβ40 or p-tau/Aβ42 ratios diagnose not only primary AD but also AD co-pathology in PSP and FTLD-tau. The investigators also found decreased p-tau/total tau and increased CSF neurofilament light chain (NfL) in FTLD-TDP compared to FTLD-tau, in line with prior studies (253), and that the FTLD (PSP) had low CSF total tau.

Thijssen et al. (254, 255) found that serum p-tau217 outperformed serum p-tau181 in differentiating AD from FTLD and healthy controls, while both markers were correlated with their CSF level, Aβ PET results, and underlying AD pathology. Previous clinical studies demonstrated that CSF total tau is increased in CBS compared to PSP or other atypical parkinsonism (256, 257); however, this could be due to underlying AD or at least co-pathology.

Considering that post-translational modifications of tau protein are different in PSP and AD (258, 259) and other tauopathies, more studies are needed to characterize PSP- and CBD-specific tau strains in CSF and specific assays are then necessary to detect these strains.

In PSP, serum NfL as a non-specific biomarker of neuronal injury is associated with disease severity and survival (260, 261) and high CSF NfL and p-tau181 predict prognosis (262). Moreover, high NfL levels in CSF or serum can differentiate atypical parkinsonian disorders from PD (263, 264). MicroRNAs and proteomic studies are currently being investigated in search of pathology-specific CSF or serum candidate markers to differentiate tauopathies (265, 266).

Tau real-time quaking induced conversion (RT-QuIC) assays are probably the most sensitive and specific single laboratory techniques for the determination of underlying pathology in every tauopathy-related clinical syndrome. Tau RT-QuIC assays for the detection of 3R and mixed 3R/4R seeds of PiD, AD, and chronic traumatic encephalopathy have already been developed (267). Recently, the range of tau RT-QuIC assays was extended to detect 4R seeds of PSP and CBD (268).

## Conclusion

Major advances in molecular biology, cryo-EM, and detailed clinical and pathologic characterization have allowed to disentangle various primary tauopathies, as well as their association with an heterogenous spectrum of phenotypic clinical presentations. Classifying the tauopathies according to the similarities of their strains using cryo-EM further supports the notion that PSP and CBD are different disorders. While this structural classification may allow to identify more specific biomarkers, it is unclear if it may allow to better understand their etiopathogenesis.

The typical phenotypic clinical presentations (RS, CBS, nfaPPA, fronto-behavioral/dysexecutive syndrome, and PAGF) lead us to suspect specific underlying pathologies, but it is still challenging to clinically differentiate PSP and CBD accurately due to their phenotypic overlap. Moreover, at present, only the RS presentation accurately predicts an underlying 4R tau pathology, usually PSP. On the other hand, it would also be difficult to differentiate CBD-CBS vs. AD-CBS without using specific AD biomarkers. However, the inclusion of exclusionary biomarkers required by the CBD clinical diagnostic criteria allows their distinction, which is critical for subjects' participation in therapeutic trials to slow disease progression and into future effective therapeutic interventions. This approach could improve the accuracy of the MDS-PSP criteria to diagnose PSP-CBS.

The discussed promising CSF biomarkers (Aβ42, Aβ42/Aβ40, total tau, p-tau 217, p-tau/Aβ42, NfL, RT- QuIC 3R, and 4R tau) and others (e.g., alpha-synuclein and future second generation tau PET ligands) will need to be validated in larger samples of pathologically confirmed cases. It would be relevant that these cases would have been prospectively and longitudinally evaluated in a cohort of subjects presenting with heterogenous phenotypes reflecting a variety of suspected underlying pathologies using detailed clinical, biofluid and imaging protocols. While we mostly discussed the use of biofluids and tau PET in view of the available literature, the role of cutaneous biopsies and other approaches will also need to be further investigated.

It remains to be understood how early these biomarkers could be identified to accurately diagnose these disorders. Of similar importance is to decrease the cost of assays and imaging to allow their use to diagnose subjects around the world. Improving the accuracy of prodromal and early clinical diagnostic criteria by inclusion of more sensitive and specific biomarkers, will allow to determine more efficiently which are the best therapeutic approaches to stop the progression of these disorders.

## Author Contributions

NO and AS prepared the first draft of the manuscript. IL wrote sections of the manuscript and provided review and critique to the content. All authors contributed to the article and approved the submitted version.

## Conflict of Interest

The authors declare that the research was conducted in the absence of any commercial or financial relationships that could be construed as a potential conflict of interest. IL is the Field Editor of Frontiers in Neurology however was not involved in the review of this manuscript.

## Publisher's Note

All claims expressed in this article are solely those of the authors and do not necessarily represent those of their affiliated organizations, or those of the publisher, the editors and the reviewers. Any product that may be evaluated in this article, or claim that may be made by its manufacturer, is not guaranteed or endorsed by the publisher.
