# Corrigendum: Clinical spectrum of tauopathies

**DOI:** 10.3389/fneur.2022.1015572

**Published:** 2022-09-14

**Authors:** Nahid Olfati, Ali Shoeibi, Irene Litvan

**Affiliations:** ^1^Department of Neurology, Faculty of Medicine, Mashhad University of Medical Sciences, Mashhad, Iran; ^2^UC San Diego Department of Neurosciences, Parkinson and Other Movement Disorder Center, San Diego, CA, United States

**Keywords:** tauopathy, movement, clinical, progressive supranuclear palsy, corticobasal, neurodegenerative, frontotemporal dementia, primary progressive aphasia

In the published article, there was an error in Table 2 as published. Subheading rows of the table, indicating the name of each criteria set, were not shown in the proper format. Additionally, a subheading row showing the title of each clinical category, including nfaPPA, svPPA, and lvPPA, under “Gorno-Tempini PPA criteria” was missing. The corrected Table 2 and its caption appear below.

**Table 2:** Standardized clinical diagnostic criteria of phenotypes related to primary tauopathies based on Movement Disorders Society Progressive Supranuclear Palsy (MDS-PSP) criteria, (91), Armstrong corticobasal degeneration (CBD) (24) criteria, Gorno-Tempini Primary Progressive Aphasia (PPA) criteria (92), and Rascovsky behavioral variant Frontotemporal Dementia (bvFTD) criteria (93).

	**Clinical syndrome**					
**Criteria set**	**RS**	**CBS**	**nfaPPA**	**bvFTD**	**PAGF**	**Tauopathies with Parkinsonism**
**MDS-PSP criteria** ^1^						
Probable	VSGP or SVS + Repeated falls or fall on pull test in first 3 years			VSGP or SVS + ≥ 3 of the following: • Apathy • Bradyphrenia • Dysexecutive syndrome • Reduced phonemic verbal fluency • Impulsivity, disinhibition, or perseveration	VSGP or SVS + Progressive gait freezing (Sudden, transient motor blocks/start hesitation, no/mild parkinsonism, levodopa resistant) in first 3 years	VSGP or SVS + one of: • Axial predominant, levodopa resistant bradykinesia and rigidity • Parkinsonism that is asymmetrical/with tremor/levodopa responsive
Possible	SVS + >2 steps backward on pull test in first 3 years	VSGP or SVS + Limb rigidity or akinesia or myoclonus + ≥1 cortical sign: • Orobuccal/limb apraxia • Cortical sensory deficit • Alien limb phenomena	VSGP or SVS + nfaPPA or PAOS		Progressive gait freezing in first 3 years	
Suggestive	Frequent mSWJs + Fall or >2 steps backward on pull test in first 3 years	Limb rigidity or akinesia or myoclonus + ≥1 cortical sign: • Orobuccal/limb apraxia • Cortical sensory deficit • Alien limb phenomena	nfaPPA or Progressive AOS	Frequent mSWJs or >2 steps backward on pull test in first 3 years + ≥3 of the following: • Apathy • Bradyphrenia • Dysexecutive syndrome • Reduced phonemic verbal fluency • Impulsivity, disinhibition, or perseveration		Axial predominant, levodopa resistant bradykinesia and rigidity or Parkinsonism that is asymmetrical/with tremor/levodopa responsive + one of: • Frequent mSWJs • Fall or >2 steps backward on pull test in first 3 years • s.o. PSP-SL • s.o. PSP-F • Levodopa resistant • Hypokinetic, spastic dysarthria • Dysphagia • Photophobia
**Armstrong CBD criteria**						
Probable	≥3 of: • Axial or symmetric limb rigidity or akinesia • Postural instability/falls • Urinary incontinence • Behavioral changes • VSGP/SVS	Asymmetric presentation of ≥2 cortical + ≥2 movement signs: **Cortical signs:** • Orobuccal/limb apraxia • Cortical sensory deficit • Alien limb phenomena **Movement signs:** • Limb rigidity • Limb akinesia • Limb myoclonus **Exclusionary criteria:** • Positive CSF, PET, or genetic AD biomarkers^2^ • Evidence of: LBD^3^/MSA^4^/ALS^5^/svPPA or nfaPPA • Structural lesion suggestive of focal cause • Granulin mutation or reduced plasma progranulin levels • TDP-43 mutations • FUS mutations ≥1 movement sign + ≥1 cortical sign Meeting no exclusionary criteria	Effortful, agrammatic speech + ≥1 of: • Impaired grammar/sentence comprehension with relatively preserved single word comprehension • Groping, distorted speech production (AOS)	≥2 of: • Executive dysfunction • Behavioral or personality changes • Visuospatial deficits		
**Rascovsky bvFTD criteria** ^6^						
Possible				Presence in the first 3 years of ≥3 of these symptoms: • Behavioral disinhibition^7^ • Apathy or inertia^8^ • Loss of sympathy or empathy^9^ • Perseverative, stereotyped or compulsive/ritualistic behavior^10^ • Hyperorality and dietary changes^11^ • Neuropsychological profile^12^		
Probable				All of below: • Meets criteria for possible bvFTD • Significant functional decline • Imaging results consistent with bvFTD, ≥ 1 of: • Frontal and/or anterior temporal atrophy on MRI or CT • Frontal and/or anterior temporal hypoperfusion or hypometabolism on PET or SPECT		
**Definite**				Meets criteria for possible or probable bvFTD + • Histopathological evidence of FTLD on biopsy or at post-mortem OR • Presence of a known pathogenic mutation		
**Gorno-Tempini PPA criteria** ^13^						
			**nfaPPA**	**svPPA**	**lvPPA**	
Clinical			At least one core feature: • Agrammatism • Effortful, halting speech with inconsistent speech sound errors and distortions (apraxia of speech) + ≥2 of: • Impaired comprehension of syntactically complex sentences • Spared single-word comprehension • Spared object knowledge	Both of the following core features: • Impaired confrontation naming • Impaired single-word comprehension + ≥3 of: • Impaired object knowledge, particularly for low frequency or low-familiarity items • Surface dyslexia or dysgraphia • Spared repetition • Spared speech production (grammar and motor)	Both of the following core features: • Impaired single-word retrieval in spontaneous speech and naming • Impaired repetition of sentences and phrases + ≥3 of: • Speech (phonologic) errors in spontaneous speech and naming • Spared single-word comprehension and object knowledge • Spared motor speech • Absence of frank agrammatism	
			**nfaPPA**	**svPPA**	**lvPPA**	
Imaging supported			• Clinical diagnosis of nfaPPA (as above) + ≥1 of: • Predominant left posterior fronto-insular atrophy on MRI • Predominant left posterior fronto-insular hypoperfusion or hypometabolism on SPECT or PET	• Clinical diagnosis of svPPA (as above) + ≥1 of: • Predominant anterior temporal lobe atrophy • Predominant anterior temporal hypoperfusion or hypometabolism on SPECT or PET	• Clinical diagnosis of lvPPA (as above) + ≥1 of: • Predominant left posterior perisylvian or parietal atrophy on MRI • Predominant left posterior perisylvian or parietal hypoperfusion or hypometabolism on SPECT or PET	
			**nfaPPA**	**svPPA**	**lvPPA**	
Definite			Clinical diagnosis of nfaPPA (as above) + ≥1 of: • Histopathologic evidence of a specific neurodegenerative pathology (e.g., FTLD-tau, FTLD-TDP, AD, other) • Presence of a known pathogenic mutation	Clinical diagnosis of svPPA (as above) + ≥1 of: • Histopathologic evidence of a specific neurodegenerative pathology (e.g., FTLD-tau, FTLD-TDP, AD, other) • Presence of a known pathogenic mutation	Clinical diagnosis of lvPPA (as above) + ≥1 of: • Histopathologic evidence of a specific neurodegenerative pathology (AD, FTLD-tau, FTLD-TDP, other) • Presence of a known pathogenic mutation	

AD, Alzheimer's disease; ALS, amyotrophic lateral sclerosis; AOS, apraxia of speech; bvFTD, behavioral variant frontotemporal dementia; CBD, corticobasal degeneration; CBS, corticobasal syndrome; CSF, cerebrospinal fluid; CT, computed tomography; FTLD, frontotemporal lobar degeneration; FUS, fused in sarcoma; LBD, Lewy body disease; lvPPA, logopenic variant primary progressive aphasia; MRI, magnetic resonance imaging; MSA, multiple system atrophy; mSWJs, macro-square wave jerks; nfaPPA, non-fluent agrammatic primary progressive aphasia; PAGF, progressive akinesia and gait freezing; PET, positron emission tomography; PSP, progressive supranuclear palsy; PSP-F, frontal variant of progressive supranuclear palsy; PSP-SL, speech-language variant of progressive supranuclear palsy; RS, Richardson syndrome; s.o., suggestive of; SPECT, single photon emission computed tomography; svPPA, semantic variant primary progressive aphasia; SVS, slow vertical saccades; TDP-43, transactive response DNA binding protein 43 kDa; VSGP, vertical supranuclear gaze palsy.

1 Exclusionary criteria for the MDS-PSP criteria include clinical, imaging, laboratory, and genetic markers of any PSP-mimics or differential diagnoses including AD, PD, other atypical parkinsonian disorders, motor neuron disease, vascular or other structural brain lesions, autoimmune encephalitis, metabolic encephalopathies, prion disease, sensory deficit, vestibular dysfunction, severe spasticity, lower motor neuron syndrome, leukoencephalopathy, normal pressure or obstructive hydrocephalus, Wilson's disease, Niemann-Pick disease type C, hypoparathyroidism, Neuroacanthocytosis, Neurosyphilis, Whipple's disease, MAPT, and other genetic mutations mimicking PSP clinically.

2 Laboratory findings strongly suggestive of AD such as low CSF Aβ42 to tau ratio or positive 11C–Pittsburgh compound B PET; or genetic mutation suggesting AD (e.g., presenilin, amyloid precursor protein).

3 Classic 4-Hz Parkinson disease resting tremor, excellent and sustained levodopa response, or hallucinations.

4 Dysautonomia or prominent cerebellar signs.

5 Presence of both upper and lower motor neuron signs.

6 Exclusion criteria: Pattern of deficits is better accounted for by other non-degenerative nervous system or medical disorders/Behavioral disturbance is better accounted for by a psychiatric diagnosis/Biomarkers strongly indicative of Alzheimer's disease or other neurodegenerative process.

8 At least one of: Socially inappropriate behavior/Loss of manners or decorum/Impulsive, rash, or careless actions.

8 At least one of: Apathy/Inertia.

9 At least one of: Diminished response to other people's needs and feelings/Diminished social interest, interrelatedness or personal warmth.

10 At least one of: Simple repetitive movements/Complex, compulsive or ritualistic behaviors/Stereotypy of speech.

11 At least one of: Altered food preferences/Binge eating, increased consumption of alcohol or cigarettes/Oral exploration or consumption of inedible objects.

12 All of: Deficits in executive tasks/Relative sparing of episodic memory/Relative sparing of visuospatial skills.

13 Inclusion criteria: most prominent clinical feature is difficulty with language; these deficits are the principal cause of impaired daily living activities; aphasia should be the most prominent deficit at symptom onset and for the initial phases of the disease. Exclusion criteria: none of these criteria apply: pattern of deficits is better accounted for by other non-degenerative nervous system or medical disorders; cognitive disturbance is better accounted for by a psychiatric diagnosis; prominent initial episodic memory, visual memory, and visuoperceptual impairments; prominent, initial behavioral disturbance.

The authors apologize for this error and state that this does not change the scientific conclusions of the article in any way. The original article has been updated.

## Publisher's note

All claims expressed in this article are solely those of the authors and do not necessarily represent those of their affiliated organizations, or those of the publisher, the editors and the reviewers. Any product that may be evaluated in this article, or claim that may be made by its manufacturer, is not guaranteed or endorsed by the publisher.

